# Seeded Growth of Au@Cu_x_O Core–Shell Mesoporous Nanospheres and Their Photocatalytic Properties

**DOI:** 10.3389/fchem.2021.671220

**Published:** 2021-04-23

**Authors:** Gongguo Zhang, Yanyun Ma, Feng Liu, Zhibo Tong, Jingquan Sha, Wenjun Zhao, Maochang Liu, Yiqun Zheng

**Affiliations:** ^1^Department of Chemistry and Chemical Engineering, Jining University, Qufu, China; ^2^Jiangsu Key Laboratory for Carbon-Based Functional Materials & Devices, Institute of Functional Nano & Soft Materials (FUNSOM), Soochow University, Suzhou, China; ^3^International Research Center for Renewable Energy, National Key Laboratory of Multiphase Flow in Power Engineering, Xi'an Jiaotong University, Xi'an, China

**Keywords:** nanocrystal, photocatalytic degradation, copper, mesoporous, seeded growth

## Abstract

We report a facile synthesis of Au@Cu_x_O core–shell mesoporous nanospheres with tunable size in the aqueous phase *via* seeded growth. The success of the current work relies on the use of a halide-free copper (Cu) precursor and n-oleyl-1,3-propanediamine as a capping agent to facilitate the formation of a copperish oxide shell with a mesoporous structure and the presence of mixed oxidation states of Cu. By varying the amount of spherical Au seeds while keeping other parameters unchanged, their diameters could be readily tuned without noticeable change in morphology. As compared with commercial Cu_2_O, the as-prepared Au@Cu_x_O core–shell mesoporous nanospheres exhibit the higher adsorption ability, enhanced activity, and excellent stability toward photocatalytic degradation of methyl orange (MO) under visible light irradiation, indicating their potential applications in water treatment.

**Graphical Abstract:**
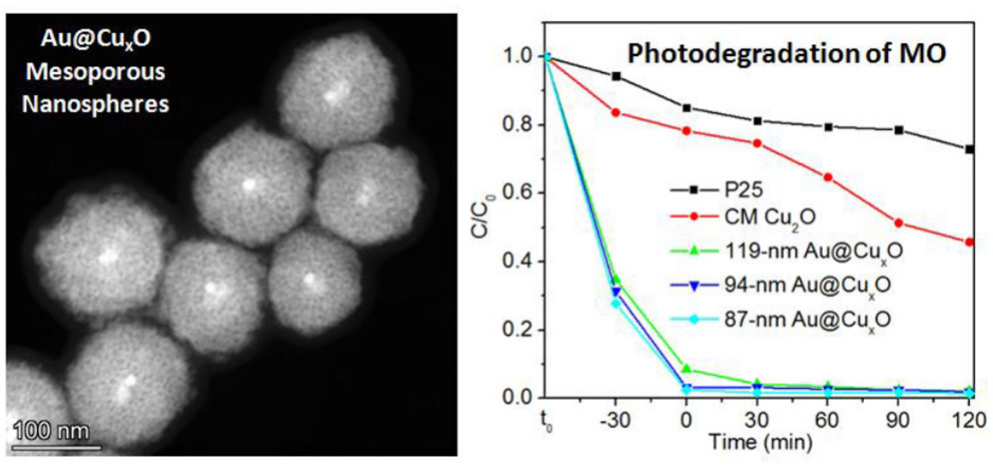
Au@CuxO core–shell mesoporous nanospheres with tunable sizes are prepared, exhibiting superior activities in photodegradation of organic dyes.

## Introduction

Metal-semiconductor core–shell nanocrystals have attracted enormous research interests in recent years due to their enhanced optical properties (Wu et al., 2004; Jin and Gao, 2009; Carbone and Cozzoli, 2010; Zhang et al., 2010). This is because the surface plasmon wave on the metal surface can be used to excite electrons of metal, transfer to the conduction band of semiconductor, and recombine with holes in the valence band (Kamat and Shanghavi, 1997; Scott et al., 2012). Also, the metal nanoparticles with well-controlled size and morphology can work as the seeding material to direct the growth of the semiconductor shell with tunable shape and structure. Such manipulation could be crucial if the physiochemical properties of the semiconductor material were highly related to their morphology or even facet dependent. Taking Au@Cu_2_O core–shell nanocrystals for example. Thanks to the research efforts from many groups. It is now possible to fabricate Au@Cu_2_O core–shell nanocrystals *via* a myriad of strategies, and the structure-performance relationship was established and investigated (Wang et al., 2008, 2011, 2016b; Pang et al., 2012b; Meir et al., 2013; Hsu et al., 2015; Huang et al., 2015; Asenath-Smith et al., 2017; Chen et al., 2018, 2019; Zhu et al., 2020b). Recent progress has allowed the plasmon mode tuning of the Au@Cu_2_O core–shell structure (Liu et al., 2012; Jiang et al., 2014; Shi et al., 2015; Zhang et al., 2016a) and explored its applications in the fields, such as photovoltaic (Oener et al., 2015) surface-enhanced Raman scattering (SERS) (Chen et al., 2017), photocatalysis (Niu et al., 2015; Lu et al., 2016; Wu et al., 2016; Lee et al., 2018; Kuo et al., 2019; Guo et al., 2021), electrocatalysis (Tan et al., 2019; Zhang et al., 2020), water splitting (Wang et al., 2017), photothermal (Wang et al., 2016a; Yang et al., 2016), and sensing (Rai et al., 2014; Chen et al., 2015; Su et al., 2018; Long et al., 2019).

Typical studies as pioneered by Huang et al. have systematically demonstrated the facet-dependent optical properties and conductive behavior of polyhedral Au–Cu_2_O core–shell nanocrystals (Kuo et al., 2011; Yang et al., 2014; Hsia et al., 2018). Recently, Wang and his coworkers have reported a wet-chemical strategy for precise tuning geometrical parameters of the Au–Cu_2_O core–shell nanoparticles. Owing to the advantage of controlled synthesis, the synergistic light absorption and scattering properties of the particles over a broad spectral range across the visible and near-infrared regions could be systematically and selectively tuned (Zhang et al., 2011). The morphology of Au@Cu_2_O core–shell nanoparticles was also found to impact the gas-sensing performance toward carbon monoxide, where the shape transformation from brick to a sphere significantly lowered the air resistance (Majhi et al., 2014). To this end, it is highly desirable to develop the rational synthesis of Au@Cu_2_O core–shell nanostructures with controlled size and morphology to realize manipulation over physiochemical properties.

Compared with counterparts with a solid interior, mesoporous structure exhibits intrinsic advantages. This is because more channels for reactant can be provided to diffuse into the catalyst body and thus allow maximized exposure of surface atoms of the catalyst, leading to the increased quantity of active sites (Lai and Koper, 2010; Lim et al., 2010; Pang et al., 2012a). The confined porous space could also increase the localized concentration of key intermediates, which helps accelerate the reaction kinetics for the following conversion. Recent studies have also indicated that the synergetic effect arising from central atoms with multiple oxidation states was found to be beneficial for the enhancement in catalysis (Zhang et al., 2016b; Zhu et al., 2020a). All these concepts contribute to the advance in the catalytic process, and the integration of these technical elements in one catalyst particle for enhancing catalytic performance should be expected.

Herein, we report a facile synthesis of Au@Cu_x_O core–shell microporous nanospheres (MPNSs), with tunable size in the aqueous phase *via* seeded growth. The success of the current work relies on the use of the halide-free copper (Cu) precursor and n-oleyl-1,3-propanediamine (OPDA) as the capping agent to facilitate the formation of a copperish oxide shell with a mesoporous structure and the presence of mixed oxidation states of Cu. The growth process was tracked and analyzed, using a scanning electron microscope (SEM) and an X-ray powder diffraction (XRD). By varying the amount of spherical Au seeds while keeping other parameters unchanged, their diameters could be readily tuned without noticeable change in morphology. The effect of the Cu precursor on product morphology was investigated. We also explored their applications as the catalyst for photocatalytic degradation of methyl orange (MO) under visible light irradiation, and they exhibited a higher adsorption capability, and an enhanced-catalytic-activity-excellent stability, as compared with commercial Cu_2_O.

## Experimental Details

### Materials

Gold (III) chloride trihydrate (HAuCl_4_·3H_2_O, 99.9%), copper chloride dehydrate (CuCl_2_·2H_2_O, 99%), copper bromide (CuBr_2_, 99%), copper nitrate trihydrate [Cu(NO_3_)_2_·3H_2_O, 99%], copper sulfate pentahydrate (CuSO_4_·5H_2_O, 99%), copper acetate monohydrate [C_4_H_6_CuO_4_·H_2_O, Cu(OAc)_2_·H_2_O, 99%], copper citrate [C_6_H_4_Cu_2_O_7_, Cu_2_(CA), 99.5%], copper acetylacetonate [C_10_H_14_CuO_4_, Cu(acac)_2_, 97%], copper(I) oxide (Cu_2_O, 97.0%), ascorbic acid (AA, 99.0%), sodium borohydride (NaBH_4_, 98%), hexadecyltrimethylammonium bromide (CTAB, 99%), and hexadecyltrimethylammonium chloride (CTAC, 97%) were all obtained from Aladdin Chemical (Shanghai, China) and used as received. n-Oleyl-1,3-propanediamine (OPDA, ≥95%) was obtained from AkzoNobel and used as received. MO (99%) was obtained from Sinopharm (Shanghai, China) and used as received. Nanosized titanium dioxide powder of P25 was purchased from Degussa and used as received. In all experiments, deionized water is used with a resistivity of 18.2 MΩ·cm, which was prepared using an ultrapure water system (Ulupure, Beijing, China).

### Standard Synthetic Procedure for Au@Cu_x_O Core–Shell MPNSs

The 10-nm, spherical Au seeds were generated, using the protocol as described in the previous study (Zheng et al., 2013a). In the following synthesis, 50 mg of Cu(acac)_2_, 200 μl of OPDA, and 200 μL of 10-nm Au seed stock solution were mixed with 10 ml of water in a 20 ml glass vial, followed by the addition of aqueous solution of AA (100 mM, 2 ml) and heating at 100°C in an oil bath for 30 min. The reaction was cooled in ice water, collected by centrifugation (16,000 rpm, 10 min), and washed with water once prior to characterization and further use.

### Photodegradation Test

Mild test conditions [room temperature, neutral solution, and simulated sunlight (500 W halide lamp; Philips)] were adopted in our experiments. The MO was used as the degraded substance to evaluate the photodegradation catalytic performance. Two milligrams of the catalysts, which was mixed with 18 ml of water containing 160 micrograms of MO and stirred for 30 min in the dark to reach adsorption equilibrium. Then, the simulated sunlight turned on with starting the clock. About 1 ml of aliquots was taken out at the time interval of 30 min, followed by filtration, using a polytetrafluoroethylene (PTFE) syringe filter, having a filtration accuracy of.2 μm and centrifugation at 16,000 rpm for 10 min to remove the catalysts. The absorbance of as-filtered solution was measured by the UV-vis-NIR spectrophotometer.

### Instrumentation

Transmission electron microscopy (TEM), high-resolution TEM (HRTEM), electron diffraction (ED), high-angle annular dark field-scanning transmission electron microscopy (HAADF-STEM), and EDX-STEM mapping were performed using a Talos F200X (FEI, United States) microscope operated at the acceleration voltage of 200 kV. Scanning electron microscopy (SEM) images were obtained using a Zeiss Ultra60 microscope operated at 12 kV. All extinction and diffuse-reflectance spectra were recorded using a T9 dual-beam UV-vis-NIR spectrophotometer (Persee, China). Nitrogen adsorption and desorption measurements were carried out with a gas adsorption instrument (3H-2000PS2, BeiShiDe Instrument, China), and the surface area of the samples was calculated according to the Brunauer–Emmett–Teller (BET) method. The pore size distribution curves were obtained by the Barrett–Joyner–Halenda (BJH) method from the nitrogen adsorption data.

## Results and Discussion

### Tracking the Formation Process

**Figure 1:**
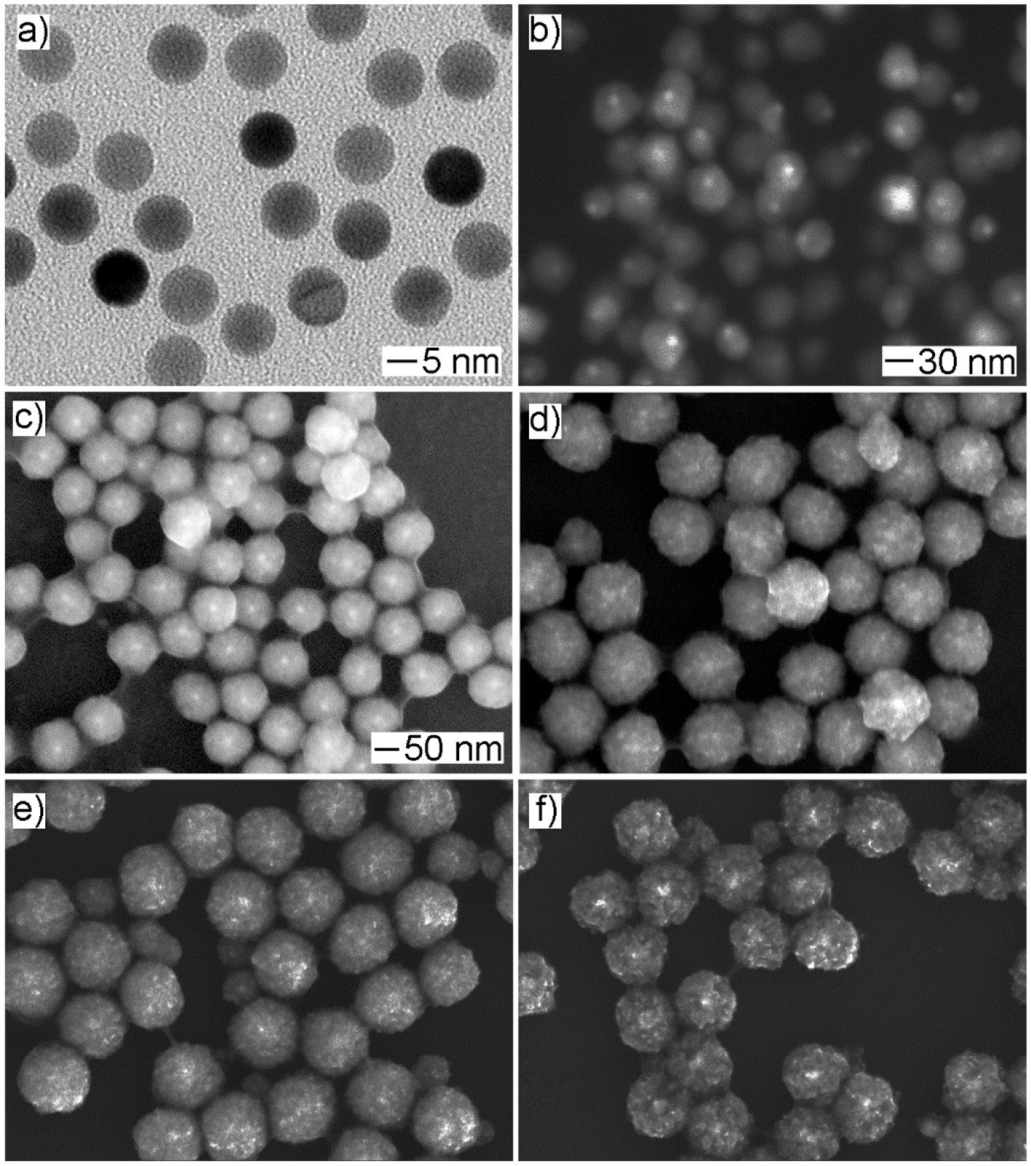
The time-lapse study of the growth process for Au@Cu_x_O core–shell nanostructures. **(a–e)** The SEM images and **(f)** the corresponding XRD patterns of the products obtained at different reaction stages of the standard procedure: **(a)** 5 min, **(b)** 15 min, **(c)** 30 min, **(d)** 45 min, and **(e)** 60 min, respectively.

**Figure 2:**
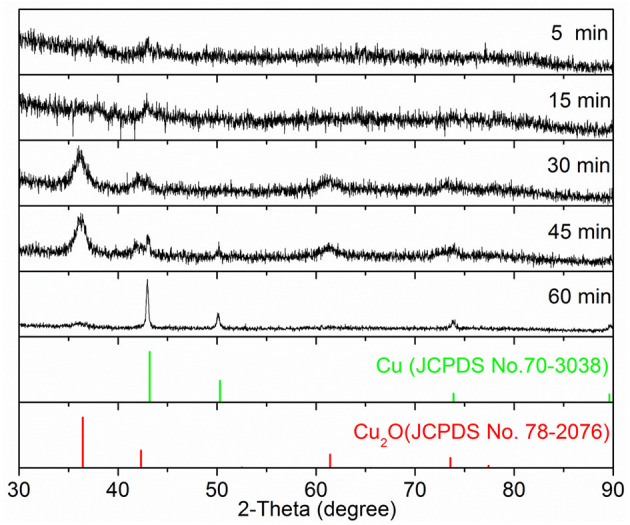
The XRD patterns of samples as displayed in Figure 1.

In a typical synthesis, the Cu precursor was reduced by ascorbic acid in the presence of spherical Au seeds and the capping agent OPDA at 100°C in the aqueous phase. To track the growth process, we terminated the reaction at different periods, followed by taking aliquots out from the reaction mixture for analysis using SEM and XRD to elucidate the evolution in morphology and structure. As shown in Figure 1, spherical nanoparticles were generated at *t* = 5 min and *t* = 15 min, and they grew in size when the reaction proceeded to 30 min. Further reaction (i.e., *t* = 45 min and *t* = 60 min) led to the formation of the products without noticeable variation in morphology but an increase in particle size. These products were also taken for XRD analysis to examine the variation in the crystal phase (Figure 2). No obvious diffraction peak could be identified for the products formed at *t* = 5 min and *t* = 15 min, which should be attributed to the poor crystallinity of the products formed at the initial stages. A diffraction peak located at two-theta of 36.4°, 42.3°, and 61.4° can be observed for the products formed at *t* = 30 min, which could be indexed to the (111), (200), and (220) crystal plane of the Cu_2_O phase (JCPDS No. 78-2076), respectively. For the products obtained at *t* = 45 min, in addition to the Cu_2_O phase, the diffraction peak at two-theta of 43.2°, corresponding to the (111) crystal plane of the Cu phase (JCPDS No. 70-3038), began to appear in the XRD pattern. Further reaction (*t* = 60 min) led to the conversion of the products fully into the Cu phase, where the decreased full width at half maxima (FWHM) of diffraction peaks suggested a better crystallinity. Taken together, these results suggested that, as the reaction proceeded, the products grew with increase in particle size and improvement in crystallinity and their crystal phase gradually converted from Cu_2_O to Cu. To this end, in order to achieve the product with a mixed crystal phase, the reaction time should be carefully controlled, and *t* = 30 min was chosen for the following synthesis (Koch et al., 2019).

### Morphology and Structure Characterization of Au@Cu_x_O Core–Shell MPNSs

**Figure 3:**
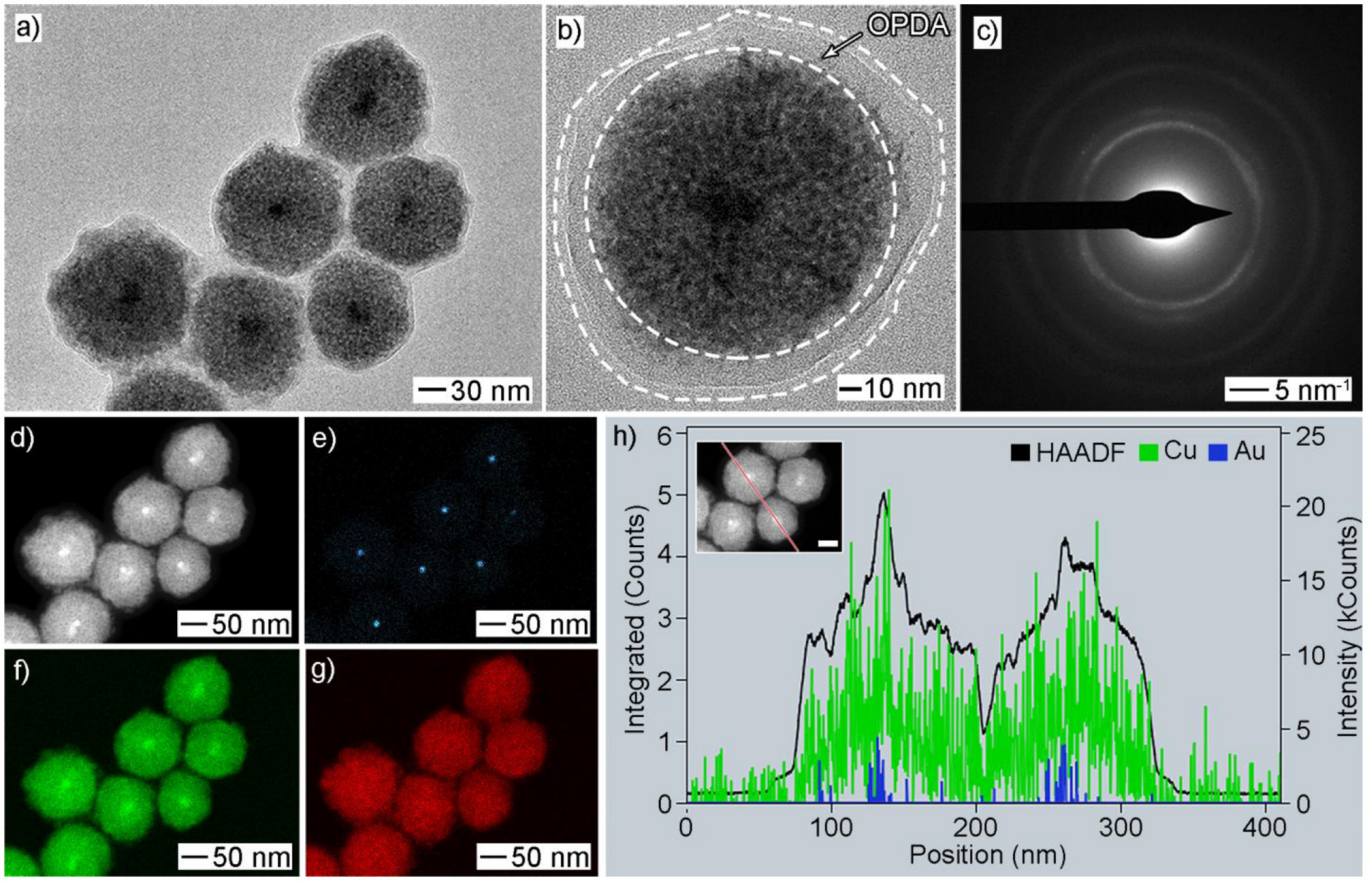
Morphology and structural characterizations of Au@Cu_x_O core–shell MPNSs as displayed in Figure 1c: **(a,b)** TEM, **(c)** ED, **(d)** HAADF-STEM, **(e–g)** EDX-mapping: **(e)** Au, **(f)** Cu, **(g)** O; **(h)** line-scan spectrum. The inset in **(e)** shows a corresponding particle with a scale bar of 50 nm.

A low-magnified SEM image of the Au@Cu_x_O products obtained at 30 min shows high uniformity in terms of size, morphology, and structure (Supplementary Figure 1). To further characterize their structure, we performed a set of characterizations, in addition to SEM, including TEM, HAADF-STEM, EDX-STEM mapping, and ED. As shown in Figures 3a,b, TEM images confirmed its core–shell and mesoporous structure. A polymer layer was noticed to be residual on the particle surface, which suggested strong covalent bonding between Cu and OPDA since these products were carefully washed with water and toluene several times. Their size was measured to be 119 ± 11 nm by statistically counting 100 typical particles (Supplementary Figure 2). Multiple diffraction circles in the ED pattern indicated their polycrystalline structure (Figure 3c). We also conducted HADDF-STEM (Figure 3d) and EDX-STEM mapping (Figures 3e–g) to investigate their elemental distribution. The EDX-STEM elemental mapping images confirmed that the elemental Au is located in the center, while elemental O is distributed in the dendritic shell. Consequently, the presence of Au seeds inside the mesoporous body could be clearly identified, demonstrating its core–shell structure. Strong intensity as a thin layer of Cu was noticed near the seed surface, indicating more Cu was distributed in this region. Figure 3h shows EDX cross-sectional line scanning, and the line profile exhibited a core–shell structure, as evidenced by the intensities of Au in the center being stronger.

**Figure 4:**
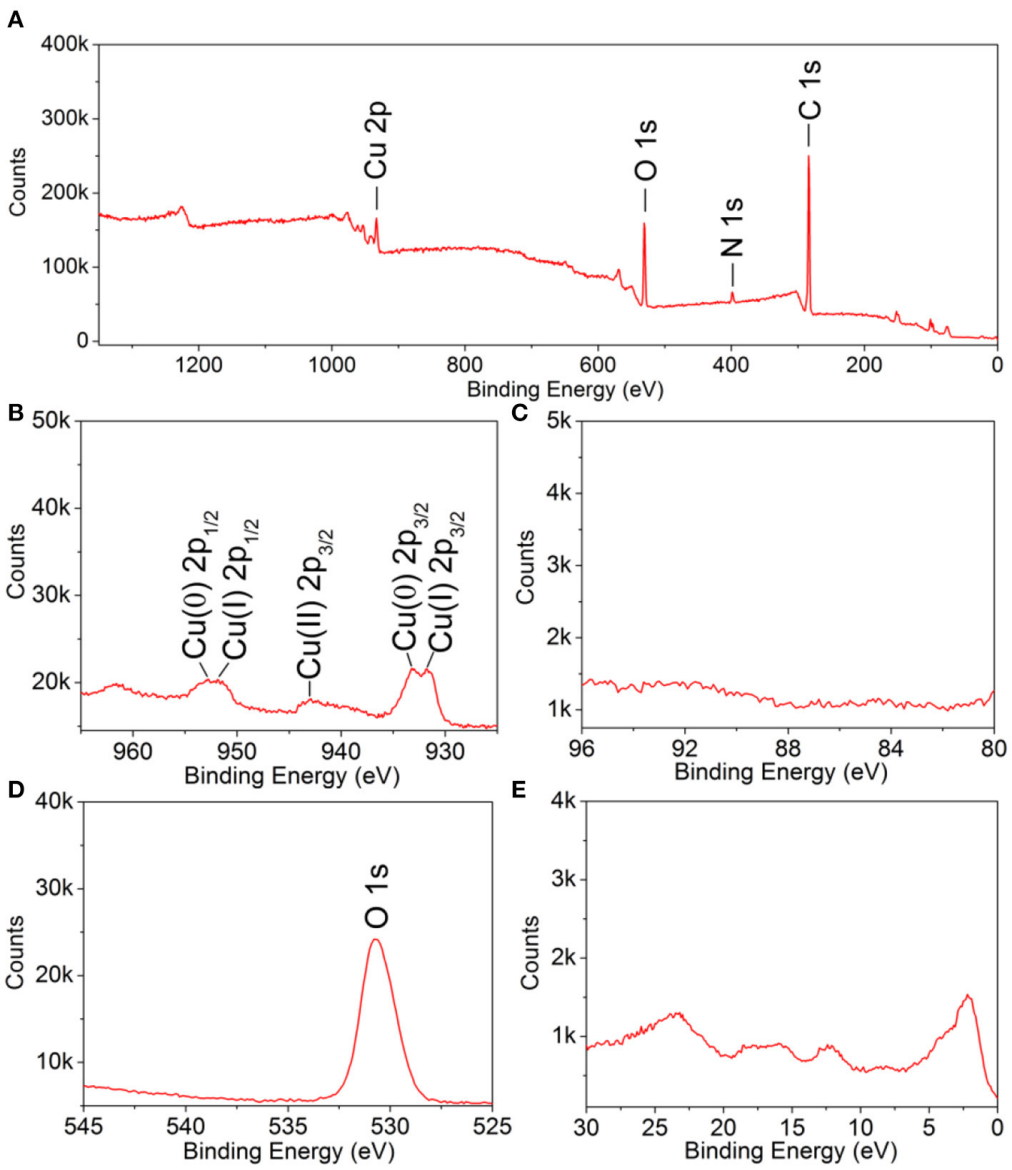
X-ray photoelectron spectroscopy spectra of 119-nm Au@Cu_x_O core–shell MPNSs: **(A)** a survey scan, **(B)** Cu 2p; **(C)** Au 4f, **(D)** O 1s, and **(E)** VB.

X-ray photoelectron spectroscopy measurements were conducted to further investigate the surface structure and electronic properties. The presence of C 1s and N 1s peaks should be attributed to the residual OPDA molecules on the particle surface (Figure 4). The strong O 1s peak indicated the presence of metal oxide. For Cu 2p, several sets of peaks were noticed: (i) the peaks located at 933.1 and 952.8 eV, respectively, corresponding to Cu(0) 2p_3/2_ and Cu(0) 2p_1/2_, respectively; (ii) the peaks located at 931.8 and 951.9 eV, respectively, corresponding to Cu(I) 2p_3/2_ and Cu(I) 2p_1/2_, respectively; (iii) the peak located at 942.9 eV, corresponding to Cu(II) 2p_3/2_. To this end, the mixed oxidation state of Cu(0), Cu(I), and Cu(II) was confirmed. The presence of Cu(0) and Cu(I) should be attributed to the crystal phase of Cu and Cu_2_O, as demonstrated by the XRD analysis, where the presence of Cu(II) should be caused by, partially, oxidation by atmospheric oxygen during sample preparation. The Au 4f peak was missing, which should be attributed to its limited amount in the products. Taken together, we can confirm the as-prepared products had a core–shell architecture, with a polycrystalline structure, as well as the composition of Au@Cu_x_O and mixed oxidation states of Cu.

### Effect of Cu Precursor on Product Morphology

**Figure 5:**
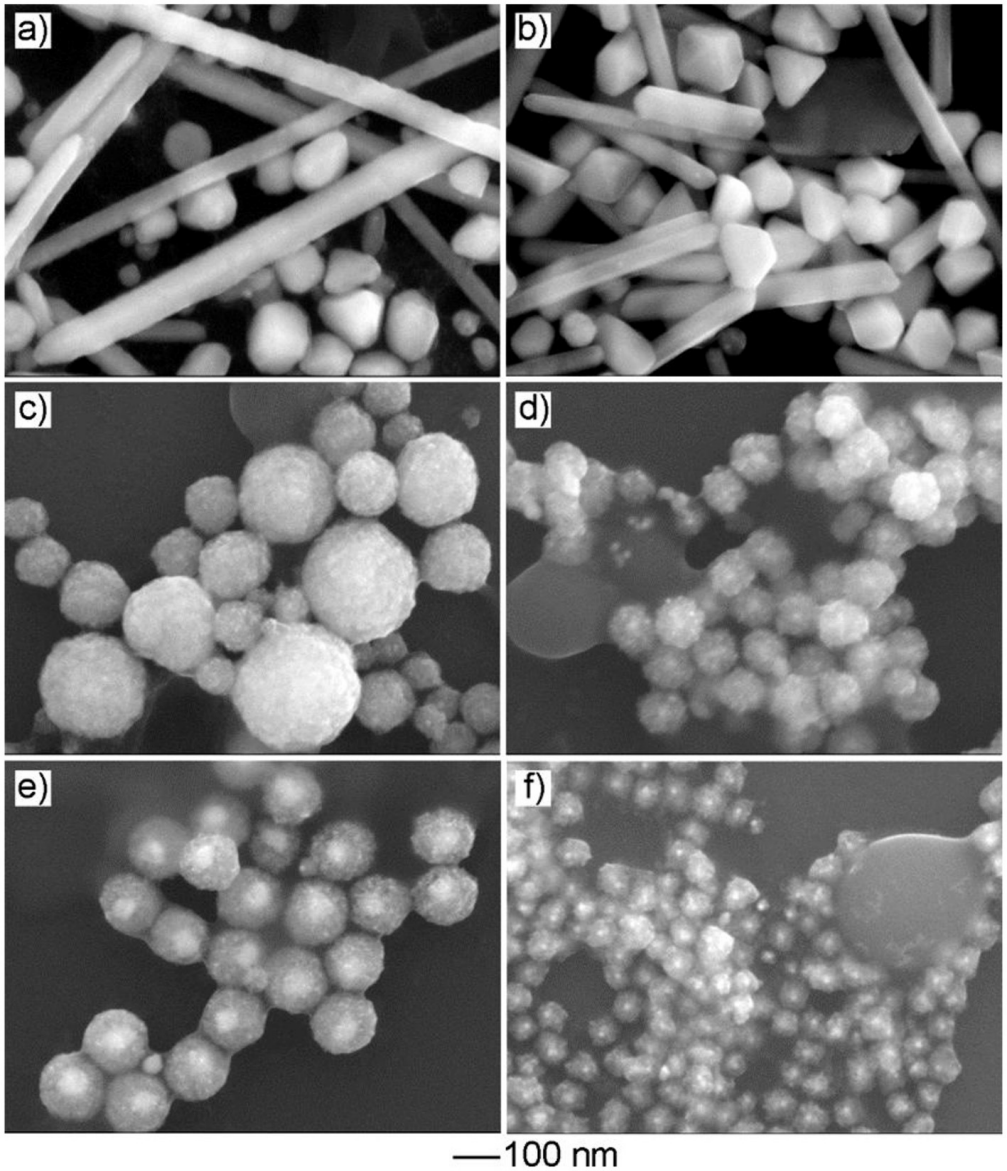
The effect of the Cu precursor on product morphology. The SEM images of the products obtained *via* the standard procedure, except that the Cu precursor was replaced by: **(a)** CuCl_2_, **(b)** CuBr_2_, **(c)** Cu(NO_3_)_2_, **(d)** CuSO_4_, **(e)** Cu(OAc)_2_, and **(f)** Cu(CA)_2_, respectively.

In the current work, Cu(acac)_2_ was employed as the Cu precursor. To evaluate the effect of the precursor on product morphology, we tried to reconduct the standard procedure by replacing Cu(acac)_2_ with other types of Cu salts or coordination compounds while keeping other conditions unchanged. As shown in Figures 5a,b, the use of the Cu precursor with halide ions, such as CuCl_2_ and CuBr_2_, led to the formation of products with a solid interior as well as well-defined polyhedral morphologies, such as cube, right bipyramid, and pentagonal rod/wire. In contrast, the use of the other types of the halide-free Cu precursor, such as Cu(NO_3_)_2_, CuSO_4_, Cu(OAc)_2_, and Cu(CA)_2_, resulted in the generation of the products with similar mesoporous structure, but the particle size varied (Figures 5c–f). These results indicated that the absence of halide ion in the reaction solution was crucial to the formation of the products with a mesoporous structure. As indicated in the previous study, halide ion can cooperate with atmospheric oxygen to form an etchant pair and such an oxidative etching can effectively remove twinned particles during the synthesis of noble-metal nanocrystals. Compared with the polyhedral counterparts, the products with a mesoporous structure had a large number of defective regions in the particle, and these regions were sensitive and vulnerable under oxidative etching environment (Zheng et al., 2013b). To this end, the presence of halide ions would play a negative role in their successive growth and stable existence in the final products.

### Effect of Capping Agent on Product Morphology

Compared with aliphatic amine used in the previous studies, such as hexadecylamine and oleylamine, OPDA has a similar molecule as that of oleylamine, except for an extra amine group. The increase in molecular polarity can help the capping agent stabilize the Cu-based nanoparticle with improved colloidal stability in the aqueous phase. To investigate the effect of the capping agent, we tried to conduct experiments by replacing the capping agent OPDA with oleylamine and octadecylamine, respectively while keeping the other reaction parameters unchanged. As shown in Supplementary Figure 3, the resultant products no longer exhibited a mesoporous structure while tiny particles were obtained. It could be attributed to the fact that OPDA has better colloidal stability in water, as well as a stronger coordination strength with Cu to impact its reduction kinetics.

### Tuning the Size of Au@Cu_x_O Core–Shell MPNSs

**Figure 6:**
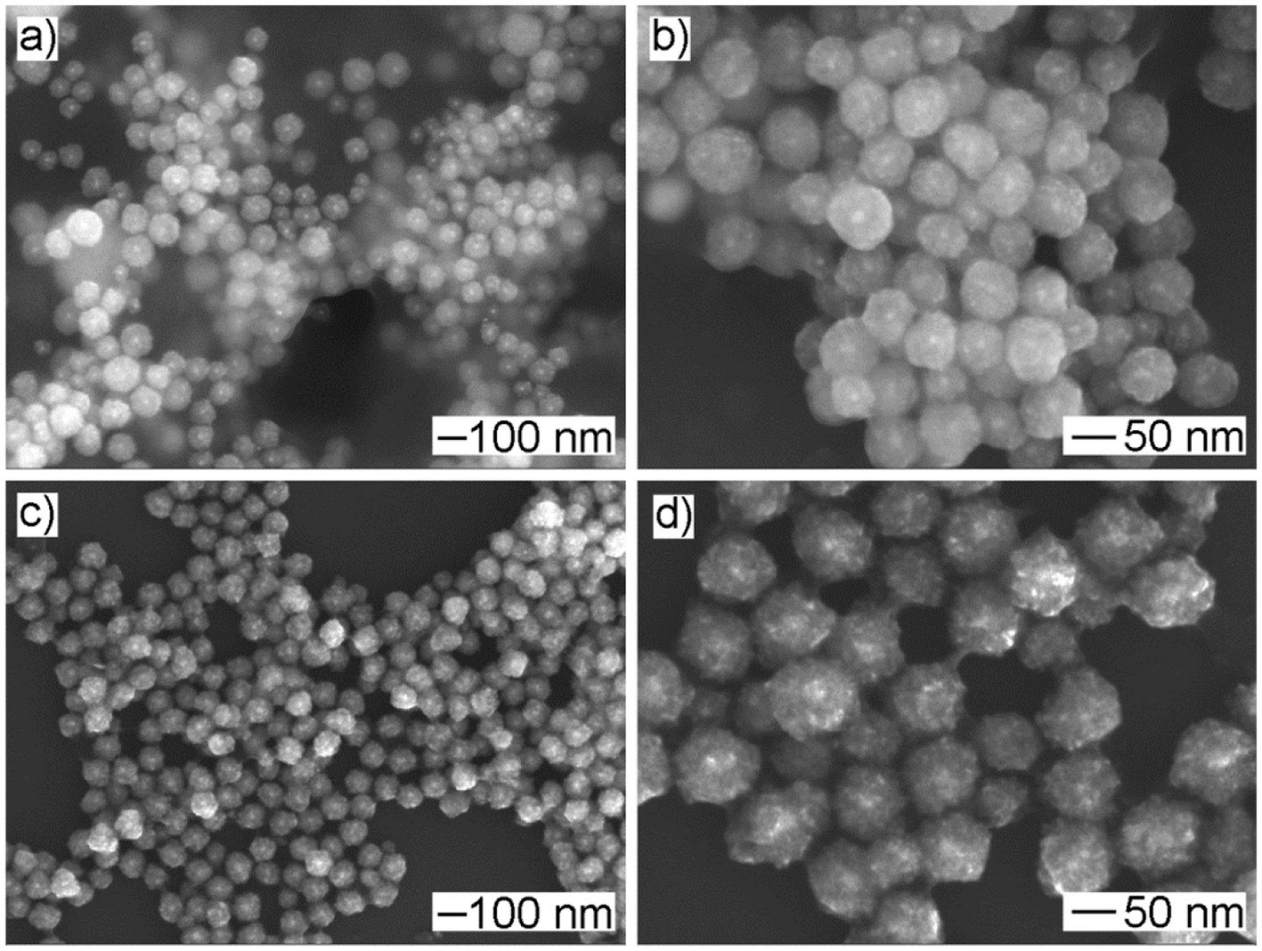
**(a,c)** Low- and **(b,d)** high-magnified SEM images of products obtained *via* the standard procedure, except that the volume of Au seed stock solution was set to **(a,b)** 1,000 μl and **(c,d)** 500 μl, respectively.

As an advantage of seeded growth, the diameter of resultant Au@Cu_x_O core–shell MPNSs could be readily tuned by simply varying the volume of the Au seed stock solution. In particular, as shown in Figure 6, the resultant products kept the morphology of MPNS without noticeable variation, but the diameter was reduced to 94 ± 10 and 87 ± 22 nm, respectively, when the volume of Au seed stock solution was increased to 500 and 1,000 μl, respectively. Their diameter distribution information was provided in Supplementary Figures 4, 5 by statistically counting 100 typical particles in corresponding SEM images. Corresponding XPS spectra confirmed that, despite the change in size, the resultant products still exhibited the presence of Cu with multiple valent states (Supplementary Figures 6, 7).

### Photocatalytic Degradation

It is well-accepted that Cu_2_O-based nanostructures can work on the effect of a catalyst toward photocatalysis. We selected the photodegradation of MO under visible light as the model reaction and employed the as-prepared Au@Cu_x_O core–shell MPNSs with different sizes to test their performance in applications of photocatalysis. Commercial Cu_2_O powder and commercial TiO_2_ nanoparticles of P25 were employed as the reference materials. The corresponding SEM image shows a broad size range of 20 nm−2 μm and quasispherical particle shape (Supplementary Figure 8). According to Beer–Lambert law, total concentrations of MO are simply determined, using the value of absorbance at λ = 464 nm as measured *via* UV-vis extinction spectroscopy. C/C_0_ was used to describe the degradation (C and C_0_ refer to mass concentrations of MO at time of *t* and *t*_0_, respectively). On the basis of the above batch experiments, the suspensions were magnetically stirred in the dark for 30 min to establish adsorption/desorption equilibrium of MO on the catalysts prior to photocatalytic tests.

**Figure 7:**
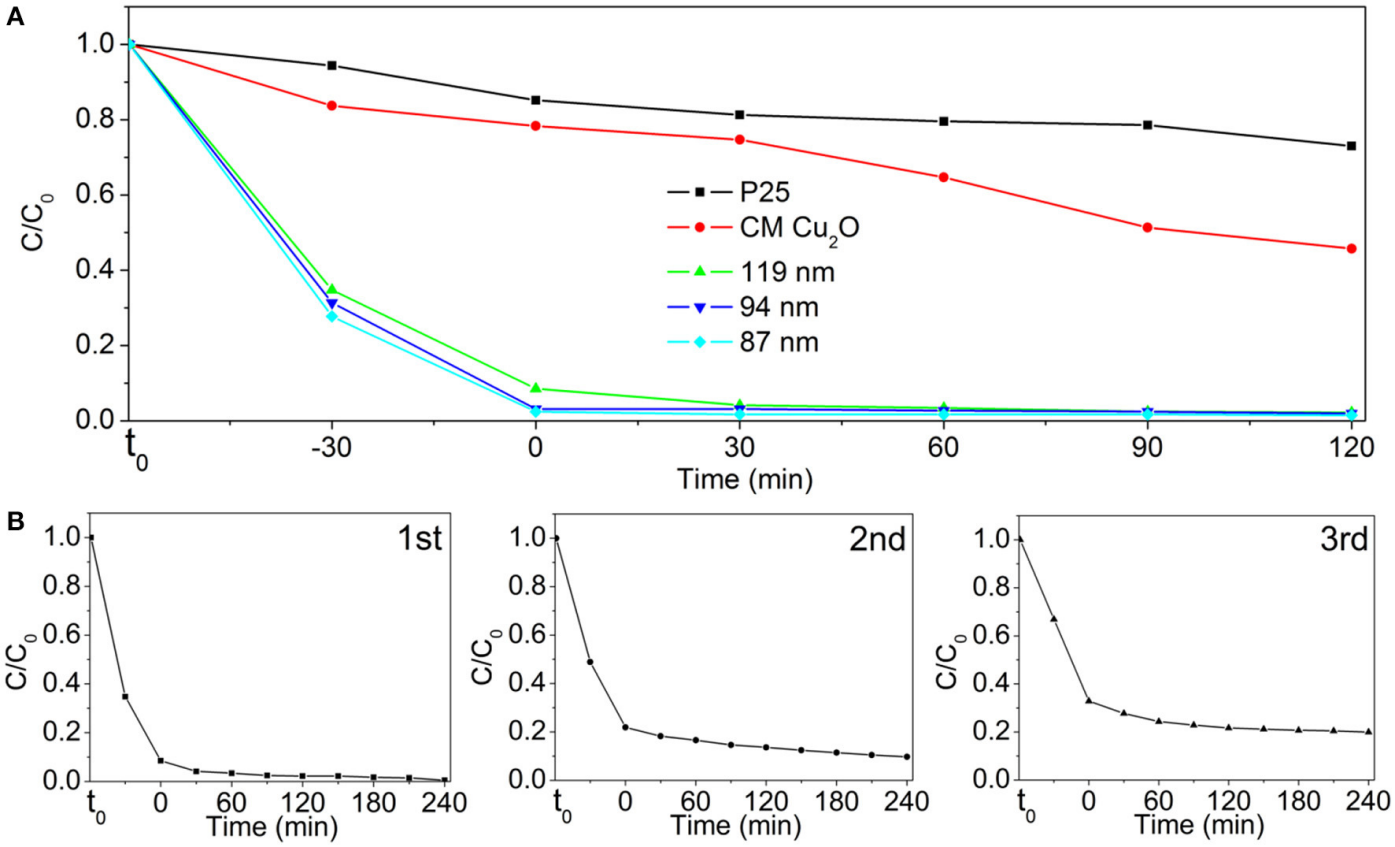
**(A)** Photodegradation of MO over P25, commercial Cu_2_O (CM Cu_2_O), and as-prepared Au@Cu_x_O core–shell MPNSs with different diameters under visible light irradiation. **(B)** Cycling runs in the photodegradation of MO in the presence of 119-nm Au@Cu_x_O core–shell MPNSs. t_0_ refers to the time point at which no catalyst is added to MO dispersion yet.

The comparison of photocatalytic activity of as-prepared Au@Cu_x_O core–shell MPNSs, commercial Cu_2_O, and P25 NPs is shown in Figure 7A and Supplementary Figures 9–13. We noticed that right after the addition of catalysts, the C/C_0_ significantly dropped for the groups using Au@Cu_x_O core–shell MPNSs (34.8, 31.4, and 27.7% for 119-, 94-, and 87-nm Au@Cu_x_O core–shell MPNSs, while 83.7 and 94.4% for commercial Cu_2_O and P25), indicating that they had a rapid and high adsorption capability toward MO. Also, the Au@Cu_x_O core–shell MPNSs display much higher photocatalytic activity than commercial Cu_2_O and P25, where about 97.8, 98.1, and 98.5% of MO molecules can be completely decomposed within 120 min for 119-, 94-, and 87-nm Au@Cu_x_O core–shell MPNSs, which are much higher than that of the other catalysts (i.e., 54.3% for commercial Cu_2_O and 27.0% for P25).

To verify the stability and reusability, cycling experiments of the 119-nm Au@Cu_x_O core–shell MPNSs catalysts were performed, where they were collected, purified, and reused in the next round of photodegradation. As shown in Figure 7B and Supplementary Figures 14–16, the 119-nm Au@Cu_x_O core–shell MPNSs for the degradation of MO exhibit a high activity and good recyclability as the conversion just a slight decline even after 12-h cycling tests. In particular, the degradation efficiency for MO was 99.5, 90.2, and 79.6%, respectively, for the consequential three cycles, respectively, confirming that the 119-nm Au@Cu_x_O core–shell MPNSs were stable during visible-light photocatalysis.

**Figure 8:**
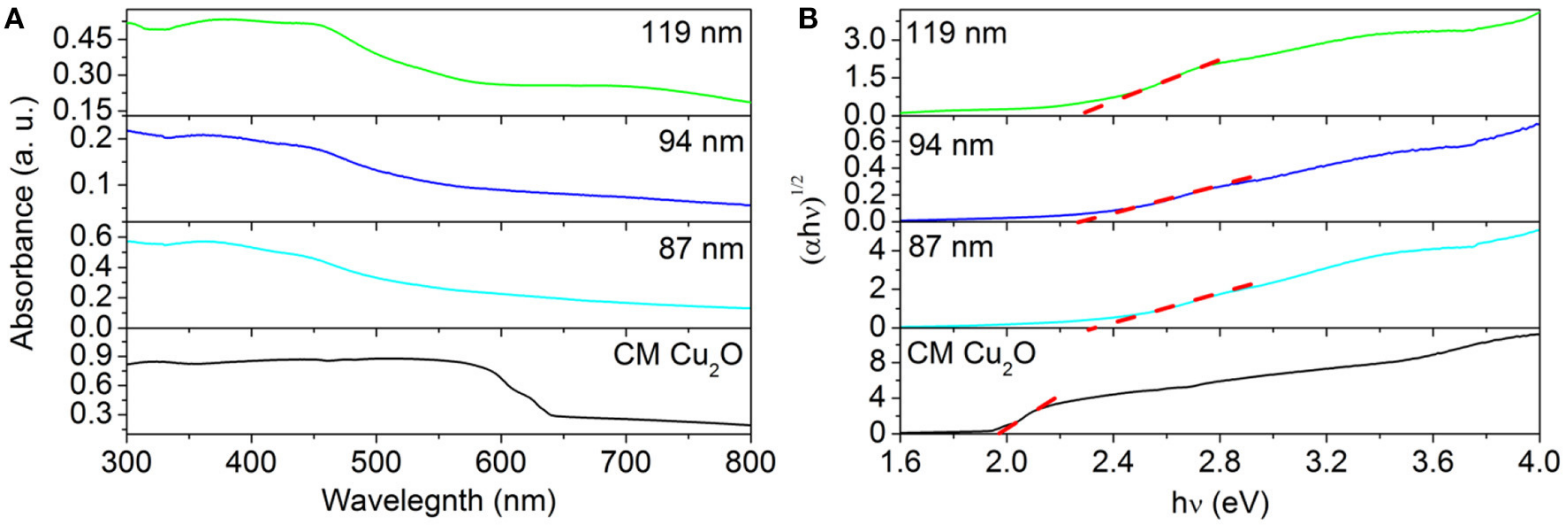
**(A)** Ultraviolet–visible spectroscopy extinction spectra of Au@Cu_x_O core–shell MPNSs with different diameters and UV-vis diffuse-reflectance spectra of commercial Cu_2_O. **(B)** Bandgap evaluation from the plots of (αE_photon_)^1/2^ vs. the energy of the absorbed light.

To investigate the mechanism responsible for the enhanced activity in the photocatalytic test, we performed a set of characterizations toward these photocatalysts to analyze their difference in the bandgap structure and the surface area. For example, UV-Vis extinction spectra of as-prepared Au@Cu_x_O core–shell MPNSs with different sizes suspended in water were recorded to investigate their optical response (Figure 8A). Unlike the red color of typical Cu_2_O powder, the aqueous suspensions of these Au@Cu_x_O core–shell were all green in color, displaying a strong absorbance in a visible range (Supplementary Figure 17). The extrapolated value (a straight line to the x-axis) of E_photon_ at at α = 0 gives absorption edge energies corresponding to E_g_, which is the bandgap of the material, which follows plots of α${\text{E}}_{\text{photon}}^{1/2}$, vs. the energy of absorbed light (where α and E_photon_ refer to the absorbance and the discrete photon energy, respectively). Figure 8B shows that the optical absorption gaps were 2.28, 2.31, and 2.36 eV for 119-, 94-, and 87-nm Au@Cu_x_O core–shell MPNSs, respectively. The UV-vis diffuse-reflectance spectrum of commercial Cu_2_O diffuse-reflectance spectrum of commercial Cu_2_O powder was also recorded, and the optical absorption gap was determined to be 1.97 eV (Jing et al., 2014). The bandgap of Au@Cu_x_O core–shell MPNSs was larger than that of commercial Cu_2_O, and it increased along with a decrease in diameter, which could be attributed to the with a decrease in diameter, which could be attributed to the quantum size effect (Ekimov et al., 1993).

**Figure 9:**
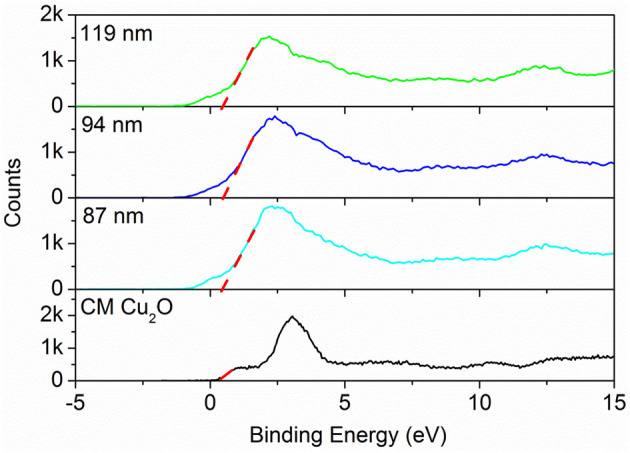
Valence band-XPS spectra of Au@Cu_x_O core–shell MPNSs with different diameters and commercial Cu_2_O.

The valence band XPS spectra (VB-XPS), near the Fermi level, were provided to explore the electronic structure of the as-prepared samples. The valence band maximum (VBM) edge potentials of Cu ion were measured by the VB-XPS spectra to demonstrate the band alignment that occurred for commercial Cu_2_O and Au@Cu_x_O core–shell MPNSs. As shown in Figure 9, the edge of VBM energy of Au@Cu_x_O MPNSs, with the diameter of 119, 94, and 87 nm, as well as commercial Cu_2_O, was determined to be about 0.38, 0.38, 0.38, and 0.33 eV, indicating that the VBM position of Au@Cu_x_O core–shell MPNSs shifted toward high-binding energy. Combined with the UV-Vis data above, the conduction band minimum (CBM) was calculated to be−1.90 eV, −1.93, −1.98, and −1.64 eV for Au@Cu_x_O core–shell MPNSs, with the diameter of 119, 94, and 87 nm, as well as commercial Cu_2_O, respectively. Herein, the higher binding energy of VBM made the valance band potential more positive, together with a wider bandgap caused by the quantum size effect, which would significantly contribute to the enhanced redox capacity and photocatalytic activity (Alivisatos, 1996; Smith and Nie, 2010).

**Figure 10:**
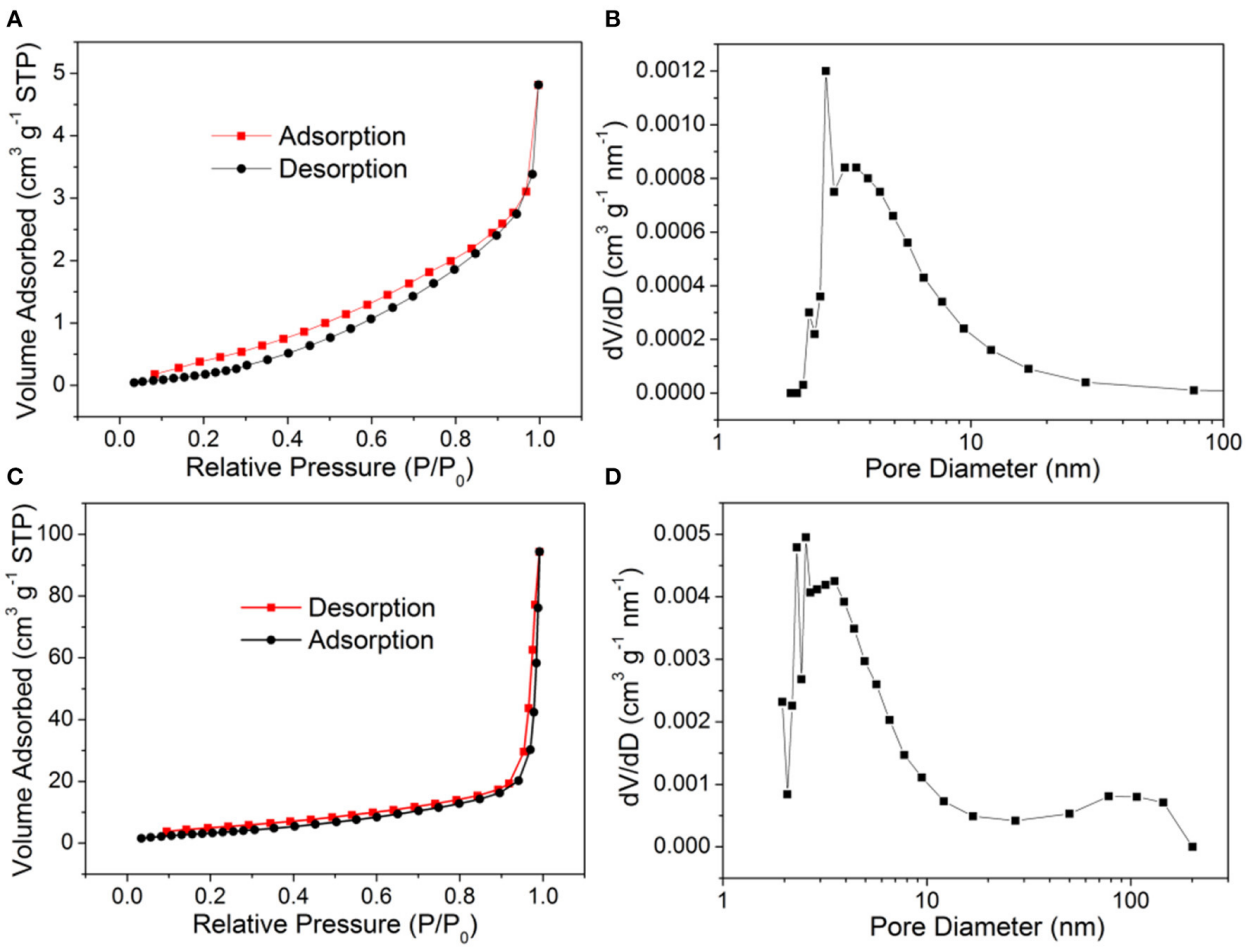
**(A,C)** N_2_ adsorption–desorption isotherms and **(B,D)** pore size distribution plots of **(A,B)** commercial Cu_2_O powder, and **(C,D)** 119-nm Au@Cu_x_O core–shell MPNSs.

In addition, nitrogen adsorption/desorption isotherms and the pore size distribution curves of 119-nm Au@Cu_x_O core–shell MPNSs and commercial Cu_2_O were recorded to illustrate the difference in the specific surface area and the porosity of the two types of the product. As shown in Figure 10, the specific surface area of commercial Cu_2_O powder and 119-nm Au@Cu_x_O core–shell MPNSs was measured to 1.97 and 14.6 m^2^g^−1^, respectively, and the corresponding average pore size was determined to be 2.67 and 2.55 nm, respectively. Compared with commercial Cu_2_O powder, the Au@Cu_x_O core–shell MPNSs show the unique mesoporous structure as well as limited particle size, which can supply a large amount of accessible surface area and active sites during photocatalysis.

## Summary

In summary, Au@Cu_x_O core–shell nanospheres with mesoporous structure and mixed valance states of Cu have been successfully prepared *via* a seed-mediated synthesis. The use of the halide-free Cu precursor and OPDA as the capping agent was found crucial to the formation of Au@Cu_x_O core–shell nanocrystals with a unique mesoporous structure. Their size could be tuned without noticeable loss in the unique morphology. Due to the advantage in structure, the current product exhibits an enhanced absorption capability and photocatalytic degradation performance toward typical organic pollutant MO. In particular, 97.8% of MO molecules can be completely decomposed within 120 min with visible light irradiation, and the degradation efficiency maintained at around 80% after a 12-h cycling test using the 119-nm Au@Cu_x_O core–shell MPNSs as the photocatalyst. The current strategy offers a facile synthesis of metal-semiconductor hybrid nanostructures with abundant active catalytic sites and could be extended to other material combinations for photocatalysis.

## Data Availability Statement

The original contributions presented in the study are included in the article/Supplementary Material, further inquiries can be directed to the corresponding author/s.

## Author Contributions

GZ: experiment, writing—original draft, and data curation. YZ: conceptualization, methodology, supervision, and writing—reviewing and editing. YM, ML, FL, WZ, ZT, and JS: formal analysis. All authors contributed to the article and approved the submitted version.

## Conflict of Interest

The authors declare that the research was conducted in the absence of any commercial or financial relationships that could be construed as a potential conflict of interest. The reviewer NL declared a past co-authorship with one of the authors ML to the handling Editor.
