# Supplementary material for: Seeded Growth of Au@CuxO Core–Shell Mesoporous Nanospheres and Their Photocatalytic Properties
Source: Front Chem. 2021 Apr 23;9:671220. doi: 10.3389/fchem.2021.671220 (PMC8103172; doi:10.3389/fchem.2021.671220)
Supplement: Supplementary file 1 [file Data_Sheet_1.docx]

Supporting Information for

**Seeded Growth of Au@Cu_x_O Core-Shell Mesoporous Nanospheres and Their Photocatalytic Properties**

Gongguo Zhang,^a^ Yanyun Ma,^b^ Feng Liu,^c^ Zhibo Tong, Jingquan Sha, Wenjun Zhao,^a^ Maochang Liu,^c^ and Yiqun Zheng^a*^

*^a^ Department of Chemistry and Chemical Engineering, Jining University, Qufu, Shandong 273115, P. R. China.*

*^b^ Institute of Functional Nano & Soft Materials (FUNSOM), Jiangsu Key Laboratory for Carbon-Based Functional Materials & Devices, Soochow University, Suzhou, Jiangsu 215123, P. R. China.*

^c^ *International Research Center for Renewable Energy, National Key Laboratory of Multiphase Flow in Power Engineering, Xi’an Jiaotong University, Xi’an, Shanxi 710049, China*

*^*^Corresponding Author:* Prof. Y. Zheng*, E-mail:* [whzyq@163.com](mailto:whzyq@163.com)

**

**

**Figure S1.** Low-magnified SEM image of 119-nm Au@Cu_x_O core-shell MPNSs.


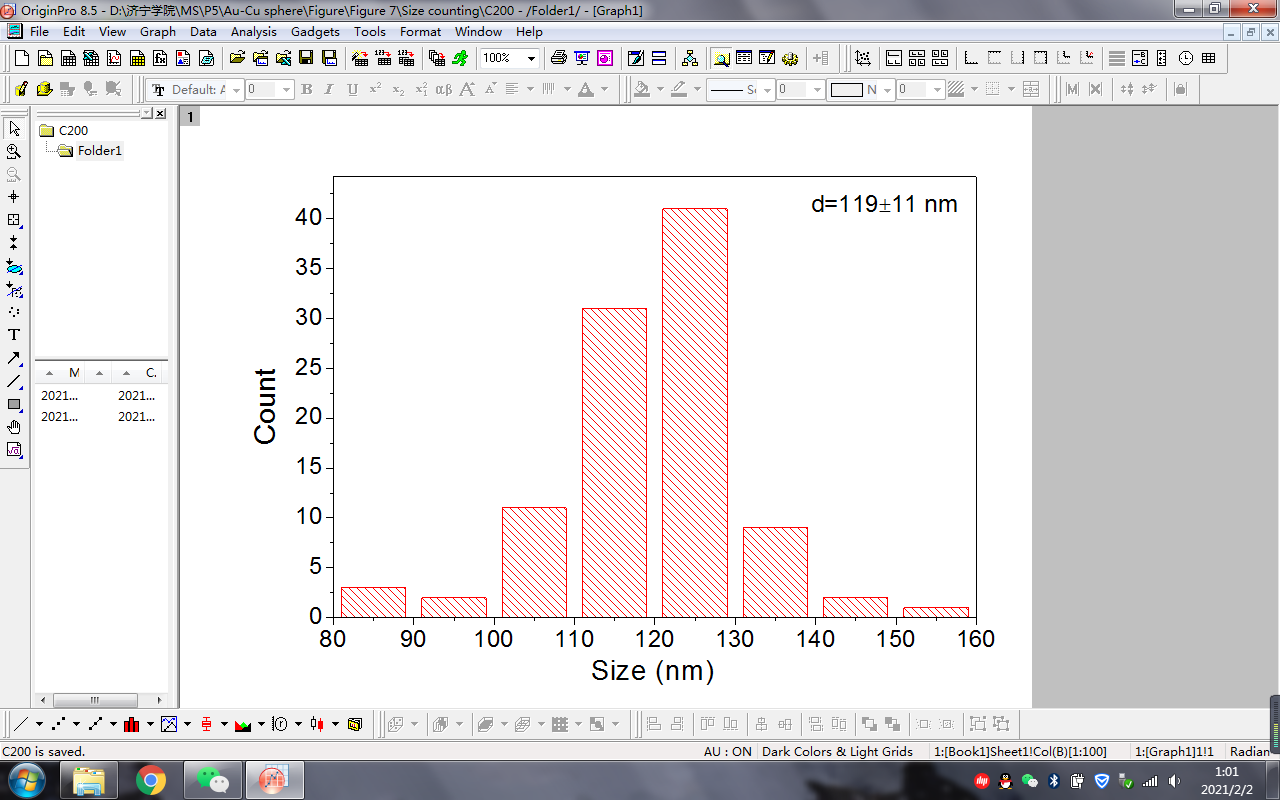


**Figure S2.** Histograms showing the diameter distribution of the Au@Cu_x_O core-shell MPNSs as shown in Figure 1c.


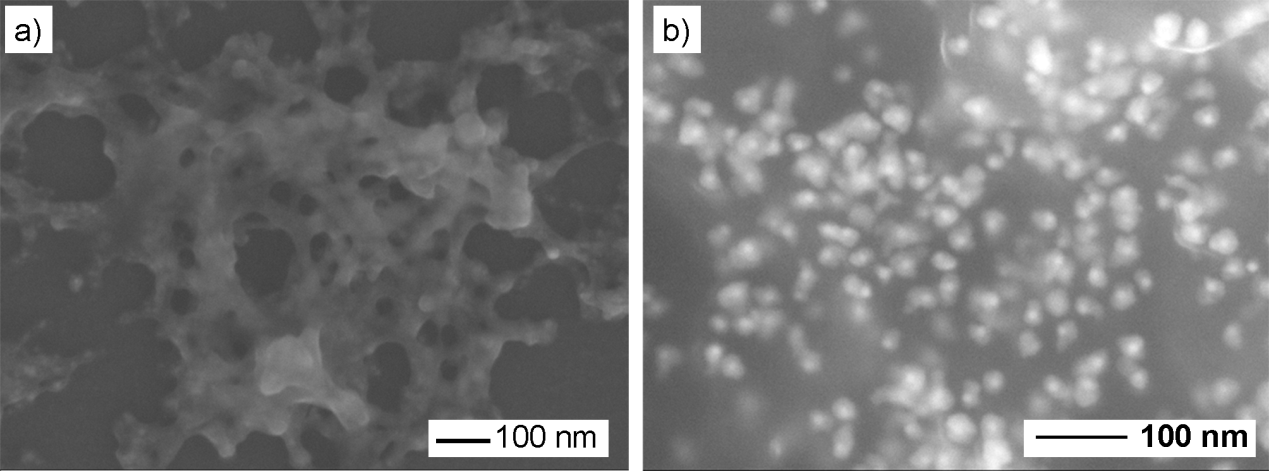


**Figure S3.** Effect of capping agent on product morphology. SEM images of products obtained via the standard procedure, except that the capping agent was replaced by: a) oleylamine and b) octadecylamine, respectively.


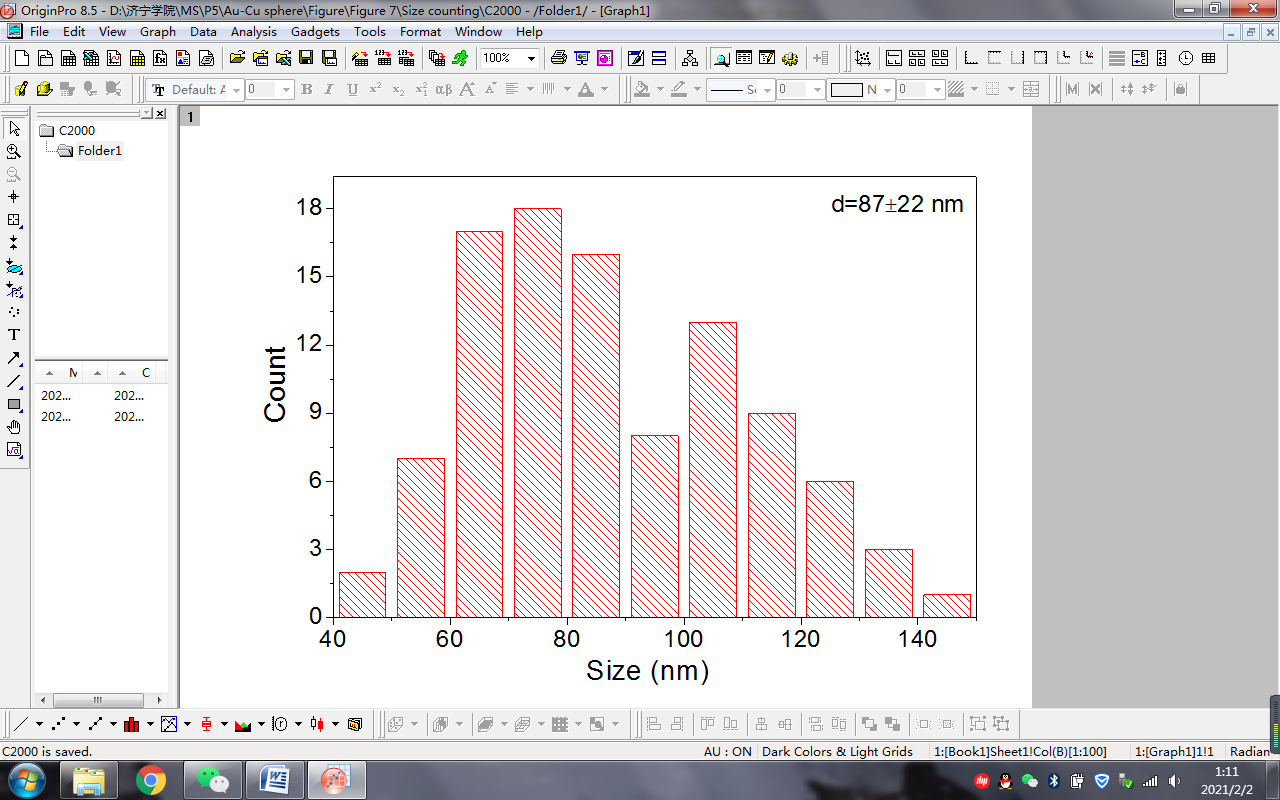


**Figure S4.** Histograms showing the diameter distribution of the Au@Cu_x_O core-shell MPNSs as shown in Figure 6, a and b.


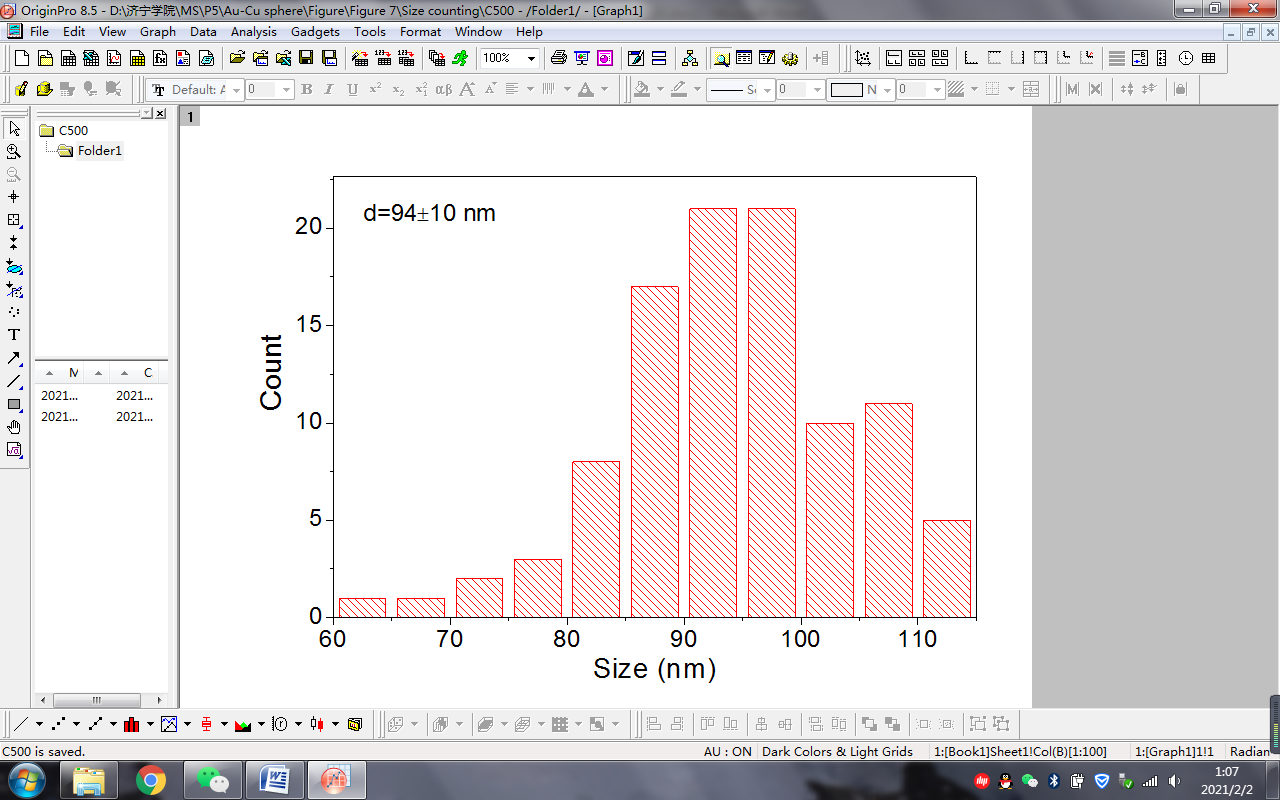


**Figure S5.** Histograms showing the diameter distribution of the Au@Cu_x_O core-shell MPNSs as shown in Figure 6, c and d.


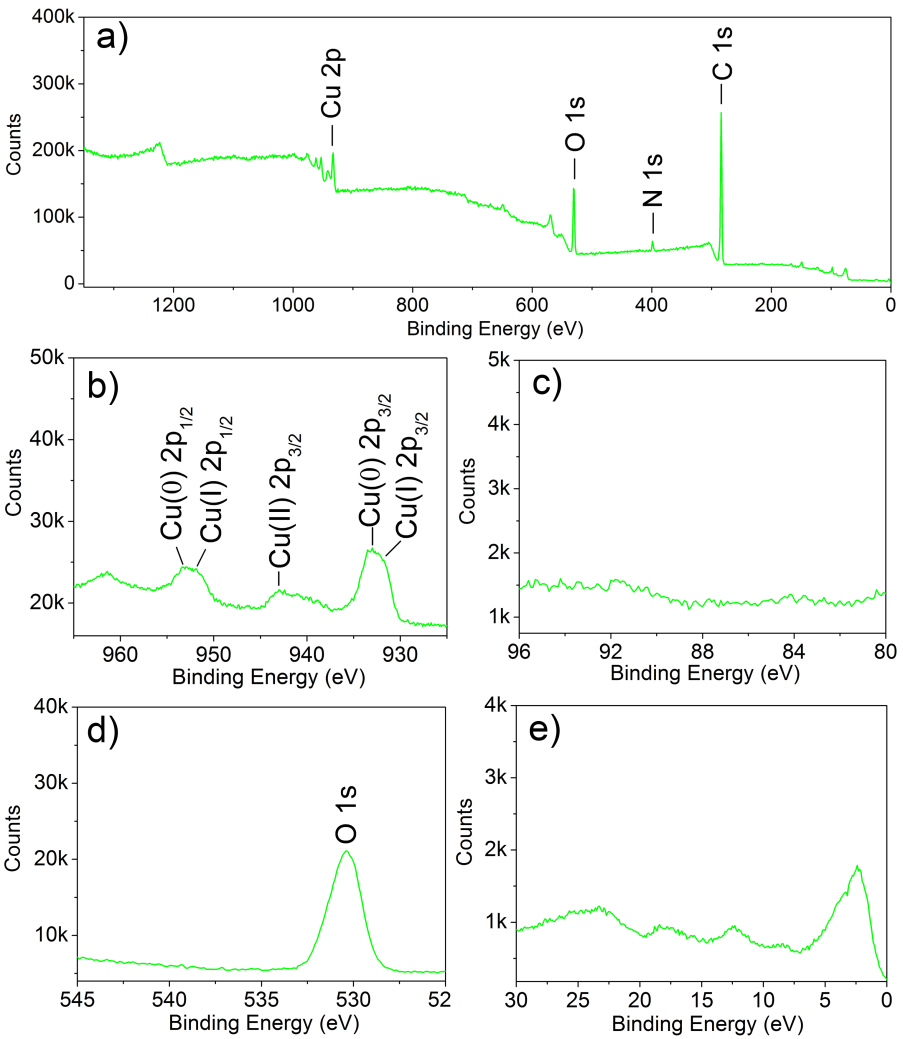


**Figure S6.** XPS spectra of 94-nm Au@Cu_x_O core-shell MPNSs: a) survey scan; b) Cu 2p; c) Au 4f; d) O 1s; e) VB.


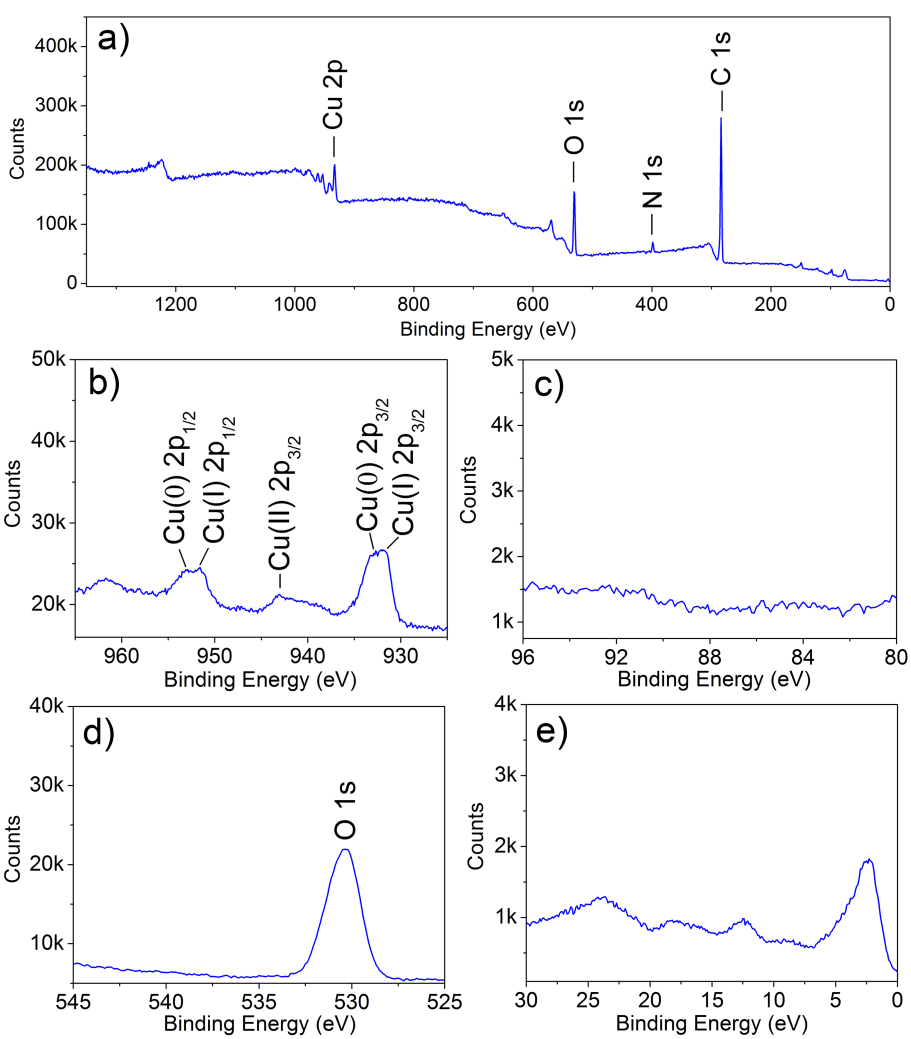


**Figure S7.** XPS spectra of 87-nm Au@Cu_x_O core-shell MPNSs: a) survey scan; b) Cu 2p; c) Au 4f; d) O 1s; e) VB.


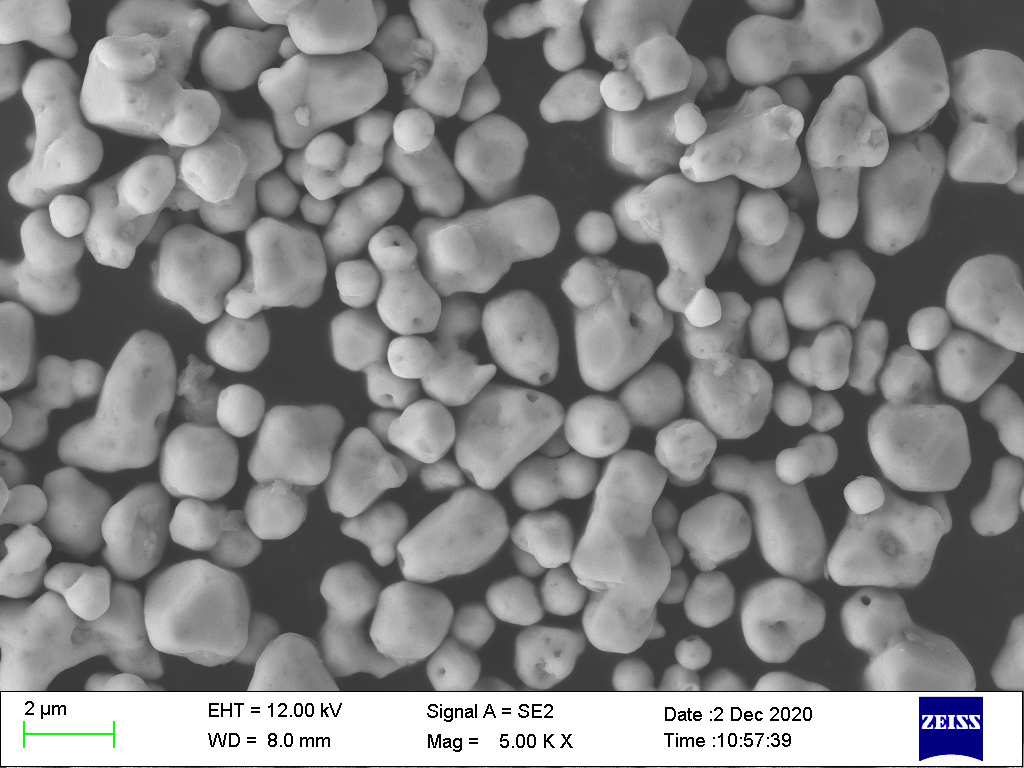


**Figure S8.** SEM image of commercial Cu_2_O powder.

**

**

**Figure S9.** UV-vis extinction spectra recorded during adsorption and photodegradtion of MO over 119-nm Au@Cu_x_O core-shell MPNSs catalyst.

**

**

**Figure S10.** UV-vis extinction spectra recorded during adsorption and photodegradtion of MO over 94-nm Au@Cu_x_O core-shell MPNSs catalyst.

**

**

**Figure S11.** UV-vis extinction spectra recorded during adsorption and photodegradtion of MO over 87-nm Au@Cu_x_O core-shell MPNSs catalyst.

**

**

**Figure S12.** UV-vis extinction spectra recorded during adsorption and photodegradtion of MO over P25 catalyst.

**

**

**Figure S13.** UV-vis extinction spectra recorded during adsorption and photodegradtion of MO over commercial Cu_2_O catalyst.





**Figure S14.** UV-vis extinction spectra recorded during the first cycle of adsorption and photodegradtion of MO over the 119-nm Au@Cu_x_O core-shell MPNSs catalyst.

**

**

**Figure S15.** UV-vis extinction spectra recorded during the second cycle of adsorption and photodegradtion of MO over the 119-nm Au@Cu_x_O core-shell MPNSs catalyst.

**

**

**Figure S16.** UV-vis extinction spectra recorded during the third cycle of adsorption and photodegradtion of MO over the 119-nm Au@Cu_x_O core-shell MPNSs catalyst.

**
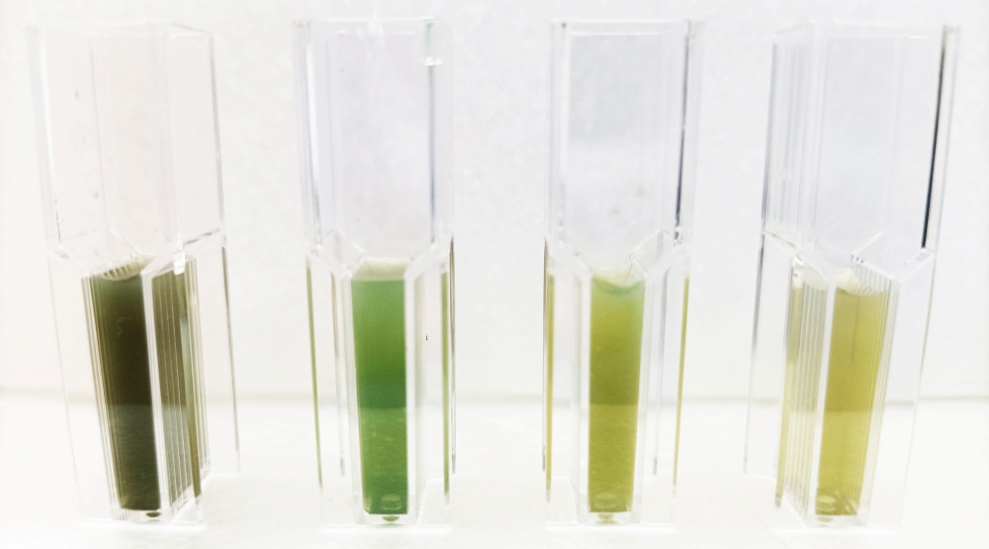
**

**Figure S17.** Digital picture shows curvets containing Au@Cu_x_O core-shell MPNSs with different diameters suspended in water: 87-nm, 94-nm, and 119-nm, from left to right.
